# The need for a structured pathway to facilitate perfusionist mobility across borders

**DOI:** 10.1051/ject/2023030

**Published:** 2023-09-08

**Authors:** Salman Pervaiz Butt

**Affiliations:** 1 Perfusionist and ECMO Specialist, Heart Vascular and Thoracic Institute, Cleveland Clinic PO Box 112412 Abu Dhabi United Arab Emirates

This letter highlights the need for a structured pathway for Perfusionists to work across borders in order to address workforce shortages and provide comparable opportunities to other medical professions. Perfusionists play a critical role in cardiothoracic surgery but face significant barriers when seeking international employment. The limited pathways can be attributed to varying recognition, the absence of an international certification framework, and countries prioritizing their own workforce planning. International collaboration is necessary to establish common standards and recognize perfusionist qualifications globally. The increasing presence of structured Perfusion education and training programs enables the establishment of a conversion pathway. Research, advocacy, bilateral agreements, and investment in training programs are recommended to address the shortage of perfusionists and enhance international mobility. A comprehensive framework with standardized curricula and guidelines would ensure consistency and quality, promoting collaboration and improving patient care. The purpose of this letter is to raise awareness and drive positive changes in international healthcare practices.

## Discussion

Limited structured pathways currently exist for skilled Perfusionists to work internationally. The recognition of Perfusion qualifications is restricted not only between countries where Perfusion is fully established and the rest of the world but also among established countries themselves. This lack of recognition hinders workforce mobility and prevents experienced Perfusionists from accessing opportunities similar to those available to doctors and nurses through established conversion routes. To address this issue and unlock new possibilities for these skilled professionals, it is essential to establish a standardized pathway that bridges the existing gap. Such a framework would not only alleviate workforce shortages but also provide Perfusionists with the means to pursue international careers and contribute their expertise on a global scale [1, 2].

Perfusionists play a critical role in Cardiothoracic surgery and Extracorporeal circulation, providing essential support to patients during surgical procedures involving the heart and lungs. Their expertise in operating heart-lung machines and other modalities including ECMO, VAD, and point-of-care testing systems is indispensable for ensuring successful outcomes. However, Perfusionists face significant barriers when seeking to work in different countries.

The limited pathways for international perfusionists to work in countries like the US, UK, Canada, and the EU can be attributed to various factors. Firstly, the recognition and demand for professions differ across countries, with nursing and medicine generally holding higher demand. Additionally, the absence of a standardized international framework for perfusionist certification and regulation creates challenges in aligning professional standards. Furthermore, countries prioritize their own workforce planning, ensuring an adequate domestic supply of healthcare professionals. As a result, the need for international perfusionists may be lower compared to other professions, leading to fewer pathways [3–6].

International collaboration among professional organizations, educational institutions, and regulatory bodies can play a vital role. By working together, these stakeholders can establish common standards and facilitate the recognition of perfusionist qualifications worldwide. A unified framework encompassing competencies, certifications, and regulations would enable perfusionists to navigate across borders more seamlessly, allowing them to contribute their skills where they are most needed.

With the increasing presence of structured Perfusion education and training programs in many countries, the establishment of a conversion pathway for Perfusionists should be readily achievable. These structured programs provide a solid foundation of knowledge and skills necessary for the practice of Perfusion. By recognizing and aligning these programs, it becomes feasible to establish a clear and streamlined pathway for Perfusionists to transition and work across borders. This recognition and harmonization of education and training standards would not only enhance the mobility of Perfusionists but also ensure a consistent level of competency and expertise in the field globally.

Furthermore, research and advocacy efforts can also help raise awareness of the value and importance of perfusionists in healthcare systems. Conducting studies to demonstrate the impact of perfusionists on patient outcomes and healthcare efficiency can garner support and recognition for the profession. Professional organizations and perfusionist associations can actively advocate for the inclusion of perfusionists in discussions surrounding international healthcare practices, ensuring their contributions are acknowledged and valued.

Bilateral agreements between countries, along with investment in training and education programs, can facilitate the movement of perfusionists across borders and address workforce shortages. These agreements would include provisions for mutual recognition of qualifications, streamlined licensing processes, and work permit arrangements, enhancing perfusionists’ mobility and career opportunities. Collaborative efforts in specialized training programs would nurture talent and alleviate regional workforce gaps. A comprehensive framework with standardized curricula, competencies, and guidelines would promote consistency, collaboration, and knowledge exchange, fostering advancements in perfusion and improving patient care on a global scale [7].

In conclusion, the establishment of inclusive pathways for perfusionists is crucial to address the current limitations in their international mobility and recognition of qualifications. By creating a standardized framework that bridges the existing gap, perfusionists can pursue international careers and contribute their expertise on a global scale. International collaboration among professional organizations, educational institutions, and regulatory bodies is key to achieving this goal. Additionally, research, advocacy, and bilateral agreements between countries can further support the inclusion of perfusionists in the global healthcare workforce, alleviating shortages and ensuring consistent quality of care.
